# Supplementary material for: Evaluating the role of common risk variation in the recurrence risk of schizophrenia in multiplex schizophrenia families
Source: Transl Psychiatry. 2022 Jul 21;12:291. doi: 10.1038/s41398-022-02060-3 (PMC9304393; doi:10.1038/s41398-022-02060-3)
Supplement: Supplementary file 1 — Supplementary Materials [file 41398_2022_2060_MOESM1_ESM.docx]

**Supplementary materials for:**

**Evaluating the role of common risk variation in the recurrence risk of schizophrenia in multiplex schizophrenia families**

Ahangari et al (2022)

Imputation quality control……………....……………....………………………………………………..2-3

Covariates…………………………………………………………………………………………6
Principal Component Analysis……………………………………………………………………………..6 Imputation quality across arrays…..…....……………....…………………………………………………..6

**Supplementary Figures:**

Supplementary Figure 1………………….………….………….………….………….………….………...3

Supplementary Figure 2…………..………….………….………….………….………….…………..……4

Supplementary Figure 3………..…………….………….………….………….………….……………......5

Supplementary Figure 4………………………………………………………………………………..…...7

Supplementary Figure 5………………………………………………………………………………..…...8

Supplementary Figure 6………………………………………………………………………………..…...9

Supplementary Figure 7………………………………………………………………………………..….10

Supplementary Figure 8………………………………………………………………………………..….11

Supplementary Figure 9………………………………………………………………………………..….12

**Supplementary Tables:**

Supplementary Table 1………….…………….………….………….………….………….……...……...12

Supplementary Table 2………….…………….………….………….………….………….………...…...13

References………………………………………………………………………………………..14

**Imputation Quality Control**

We used the following Michigan Imputation Server default quality control parameters (1):

First, chunks of 20 Mb are created from the uploaded genotypes. Then, on each chunk, the followings are checked:

1. Determine the number of valid variants in the chunk. A variant is valid when it is included in the reference panel.
2. Determine the number of variants found in the reference panel for the chunk, where at least 50% of the variants must be included in the reference panel.
3. Determine sample call rate for the chunk, where at least 50% of the variants must be called for each sample.

Chunks are excluded if number of variants < 3, overlap is < 50% and sample call rate is < 50%.

Second, the followings are checked on the variant level:

1. Only A, C, G, T alleles are allowed
2. Alternate allele frequency (AF) is calculated and all markers with AF > 0.5 are flagged.
3. SNP call rate is calculated
4. Chi square for each variant is calculated by using reference panel vs study data.
5. Allele switches are determined by comparing reference and alternate allele of the reference panel vs study data with A/T and C/G variants ignored.
6. After removing possible allele switches, strand flips are determined by flipping and comparing reference and alternate alleles from the reference panel vs study data.
7. Determine both allele switches and strand flips by combining steps 5 and 6.

Variants are excluded if alleles other than A, C, G, T are observed, duplicates are observed, indels, monomorphic sites, allele mismatch between reference panel vs study data and SNP call rate of < 90%.

Third, the followings are checked at sample level:

1. For chromosomes 1 to 22, a chunk is excluded if one sample has a call rate < 50 % with only the complete chunks excluded and not samples.
2. Liftover is performed if the build of the input data and reference panel does not match.

Following the above steps, we applied the r2 threshold of 0.3 which removed >70% of poorly imputed SNPs at the cost of < 0.5% well imputed SNPs. and minor allele frequency of 1%.

Supplementary figures 1-3 show the allele frequency correlation of arrays versus reference panel.


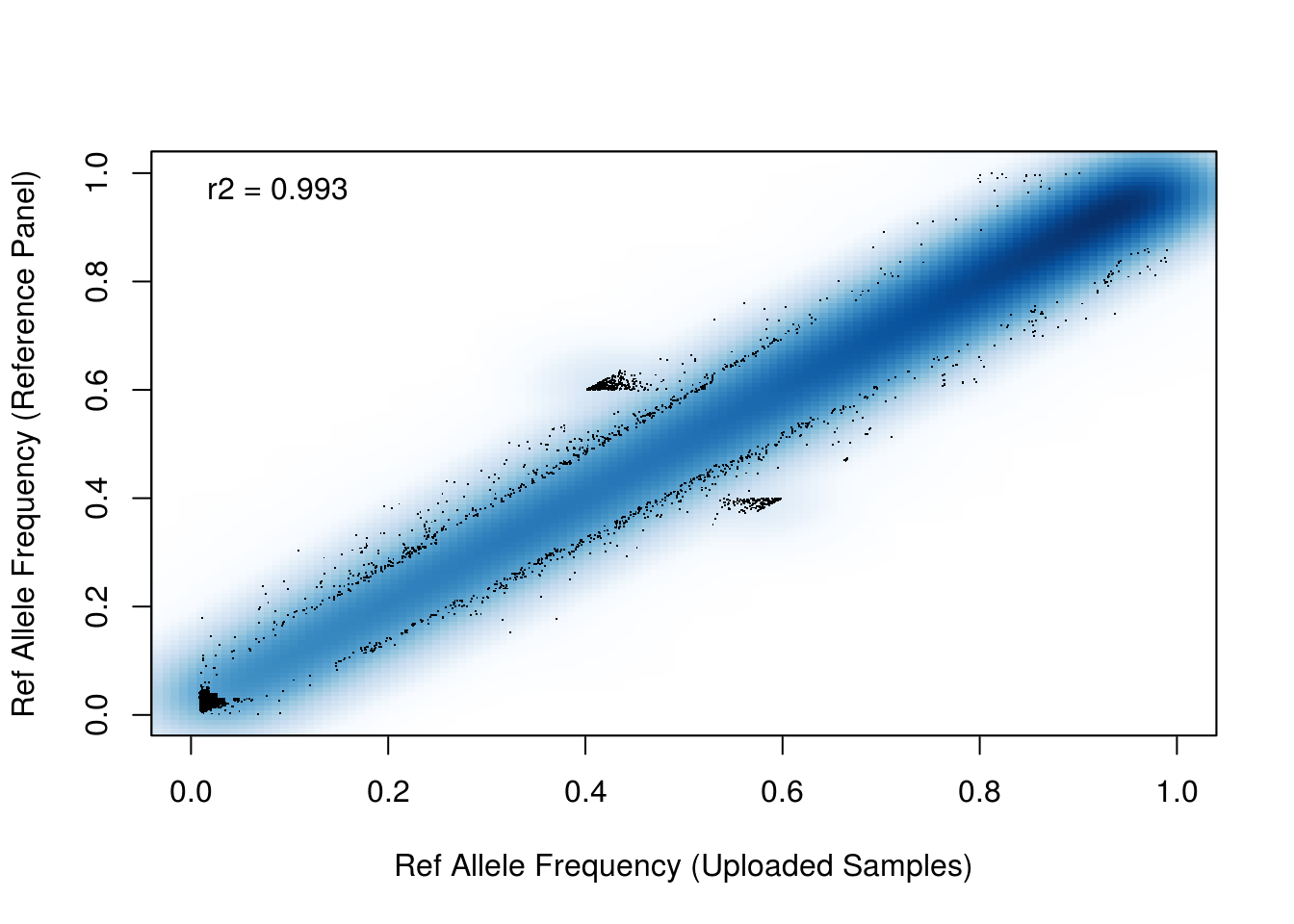


**Supplementary figure 1:** Allele frequency correlation for the Affymetrix Array vs reference panel. The plot shows the densities of frequencies falling into each part, with the first 5000 points from areas of lowest regional densities plotted.


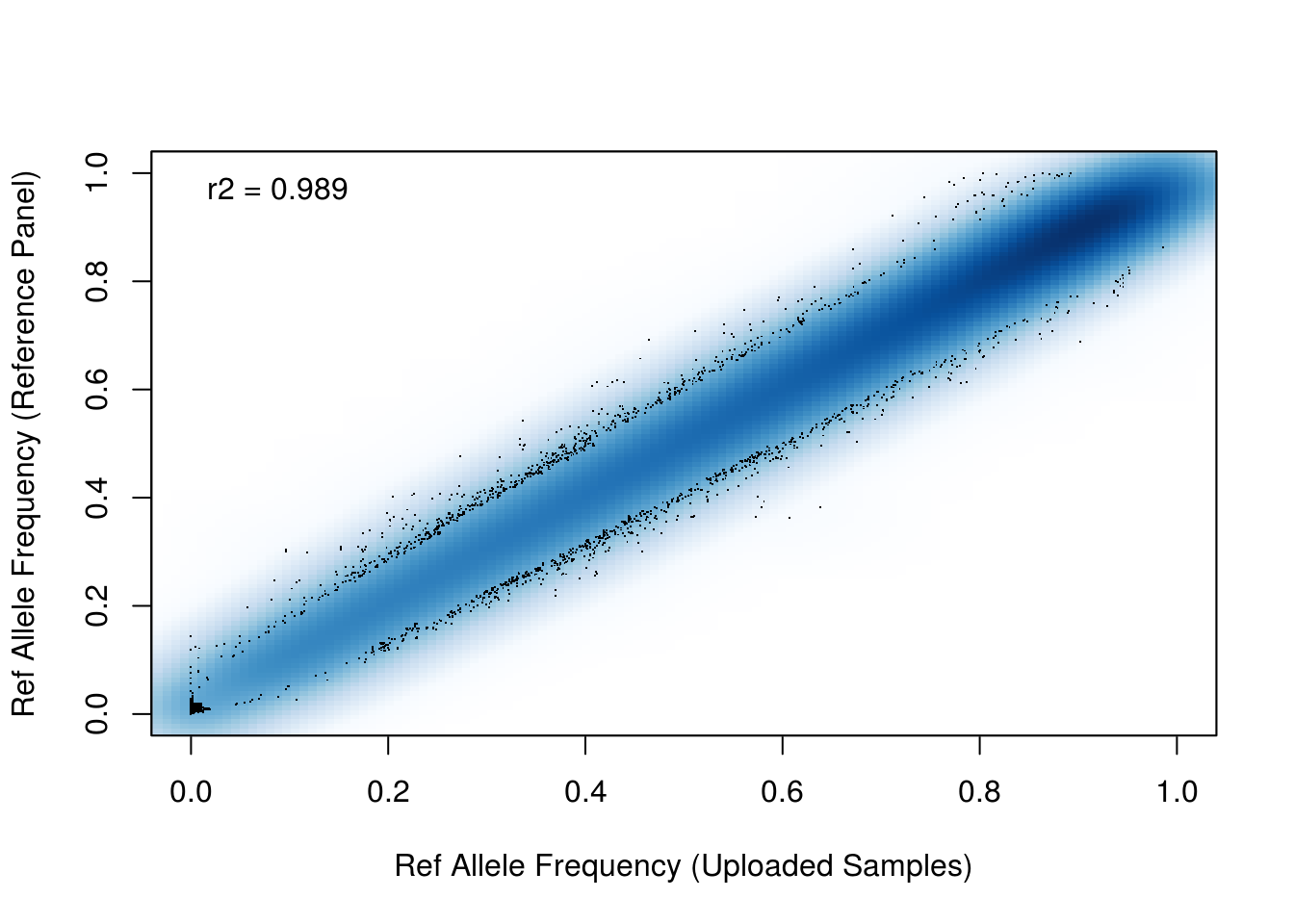


**Supplementary Figure 2:** Allele frequency correlation for the Illumina Array vs reference panel. The plot shows the densities of frequencies falling into each part, with the first 5000 points from areas of lowest regional densities plotted.


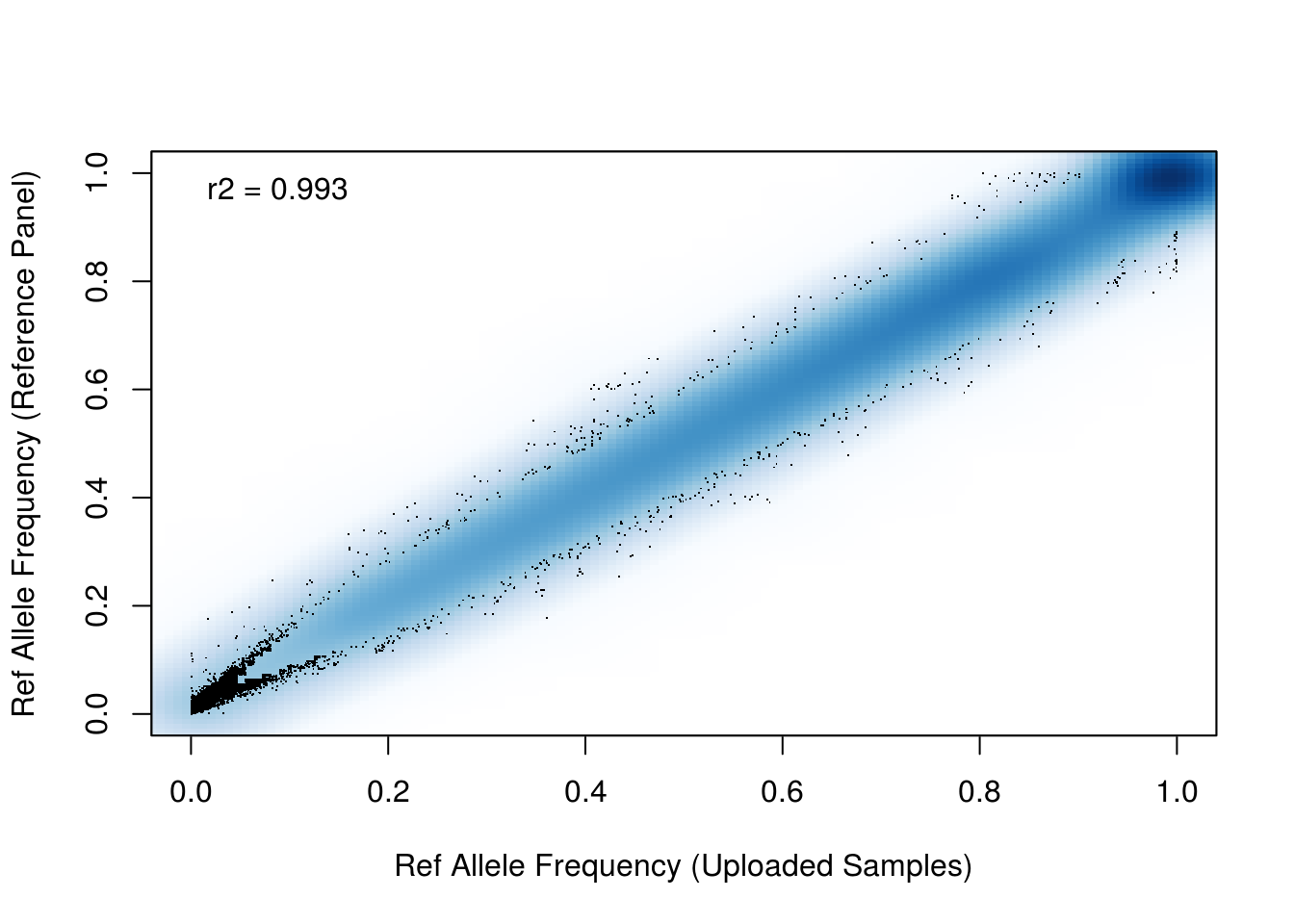


**Supplementary Figure 3:** Allele frequency correlation for the psychChip Array vs reference panel. The plot shows the densities of frequencies falling into each part, with the first 5000 points from areas of lowest regional densities plotted.

**Imputation quality across the arrays:**

After performing the QC steps described in the main manuscript under “Imputation” section in the methods, and supplemental information provided above, 9,298,012 SNPs in the Illumina Array, 11,080,279 SNPs in the Affymetrix Array, and 11,081,999 SNPs in the PsychArray remained for analysis. In total, 9,008,825 of these SNPs were shared across all three arrays. We merged these shared SNPs with HapMap3 SNPs as described in the main manuscript under “Polygenic risk score construction” section in the methods which left us with 943,020 high quality SNPs with R2 of at least 0.96 for PRS construction, as shown below:

| Array | Genotyped | Imputed | Mean R2 (SD) |
| --- | --- | --- | --- |
| Affymetrix | 443,872 | 499,148 | 0.98 (SD=0.041) |
| Illumina | 414,052 | 528,968 | 0.98 (SD=0.035) |
| PsychArray | 338,265 | 604,755 | 0.96 (SD=0.056) |

Supplementary Table1: HapMap3 SNPs across the 3 arrays used for PRS construction. Mean imputation quality scores (SD) are provided on the 4^th^ column for the imputed SNPs.

**Covariates used in the analysis:**

Subjects used in this study were genotyped on 3 different arrays as shown in the supplementary table 1. These 3 arrays are the Affymetrix V.6.0. Array, Illumina 610-Quad Array, and the Illumina Infinium PsychArray V.1.13. Case-Control samples on the Affymetrix V 6.0 array were genotyped at either the Broad Institute or Affymetrix. The ISHDSF sample were genotyped on the illumine 610-Quad array by the Illumina. Additional cases and controls and the ISHDSF sample were genotyped on the PsychArray at Mount Sinai. In order to control for possible batch or site effects, we included each of the platforms and genotyping sites as covariates. In addition, although principal component analysis (PCA) shows that all the individuals analyzed in this study are of European ancestry, due to finer-scale population structure and differences in the British Isles, we included the top 10 PCs as the covariates in our analysis.

**Principal Component analysis:**

The principal component analysis (PCA) was performed using the PLINK software (2). We used the 1000 Genomes phase 3 data set populations (3) as the background for our continental PCA analysis. Variants not on the coding from the 1000 Genomes Phase 3 dataset were first removed and only the variants with the minor allele frequency of more than 0.10 were retained. Next, all the other variants for each chromosome were pruned using the flag --indep 50 5 1.5. This left us of 349,512 variants on the 1000 Genomes Phase 3. High quality Imputed genotypes with R2 value of more than 0.99 were extracted from each of the three arrays and merged with the 349,512 variants from the 1000 Genomes Phase 3 dataset to get a list of all the available SNPs across all platforms and 1000 Genomes Phase 3 for PCA analysis. The total number of variants shared across all 3 platforms and the 1000 Genomes Phase 3 was 264,307 variants. We then used –pca flag in PLINK V.1.9 to calculate the top 20 PCAs.


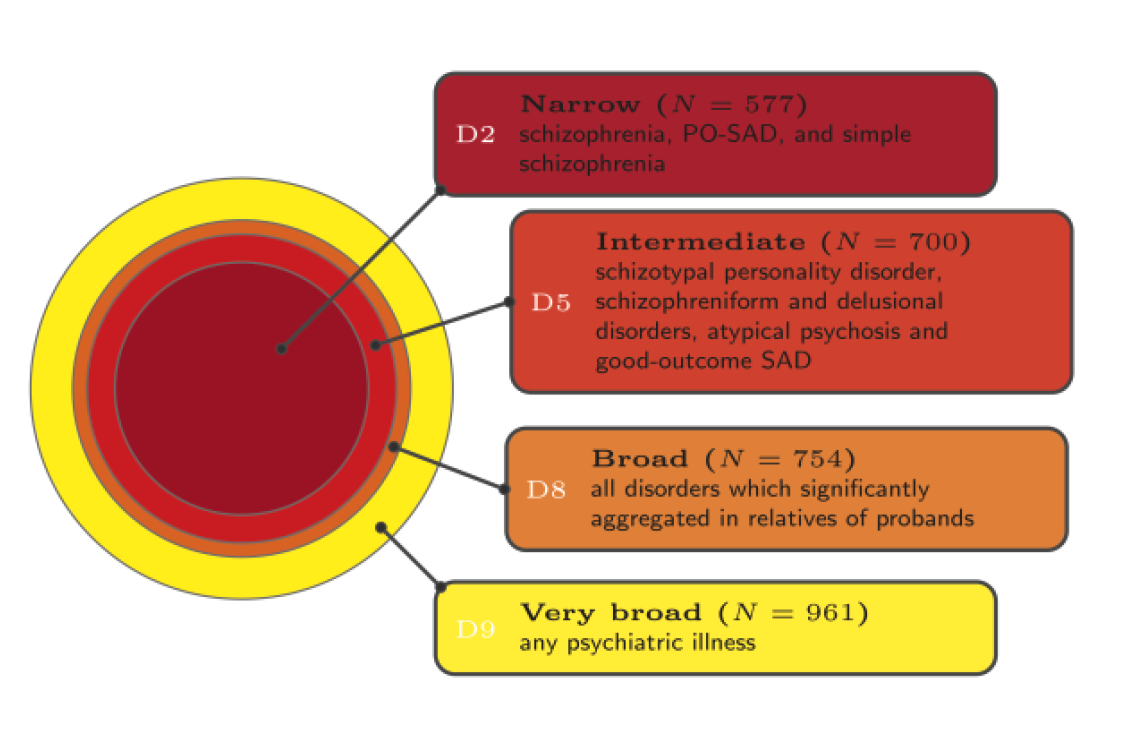


**Supplementary Figure 4:** Concentric diagnosis of ISHDSF sample. Number of individuals with phenotypic information is provided in the figure. Note that not all the individuals assessed in the ISHDSF study have available genotypes. Only those individuals with available genotypic and phenotypic information were included in the current study.

**Supplementary Figure 5:** continental PCA plot for the Affymetrix case-control samples projected on the 1000 Genomes Phase 3 data. PC1 on the X-axis and PC2 on the Y-axis. Each color represents one of the ancestries. Black color represents the Irish sample.

**Supplementary Figure 6:** continental PCA plot for the Illumina family samples projected on the 1000 Genomes Phase 3 data. PC1 on the X-axis and PC2 on the Y-axis. Each color represents one of the ancestries. Black color represents the Irish sample.

**Supplementary Figure 7:** continental PCA plot for the psychArray case-control and family samples projected on the 1000 Genomes Phase 3 data. PC1 on the X-axis and PC2 on the Y-axis. Each color represents one of the ancestries. Black color represents the Irish sample.

**Supplementary Figure 8:** fine-scale PCA analysis of the Irish population. The fine-scale population structure captured for PC1 on the X-axis and PC2 on the Y-axis represents the populations of the British Isles. Each color represents one of the arrays. The top 10 PCs were used as covariates in the analyses.


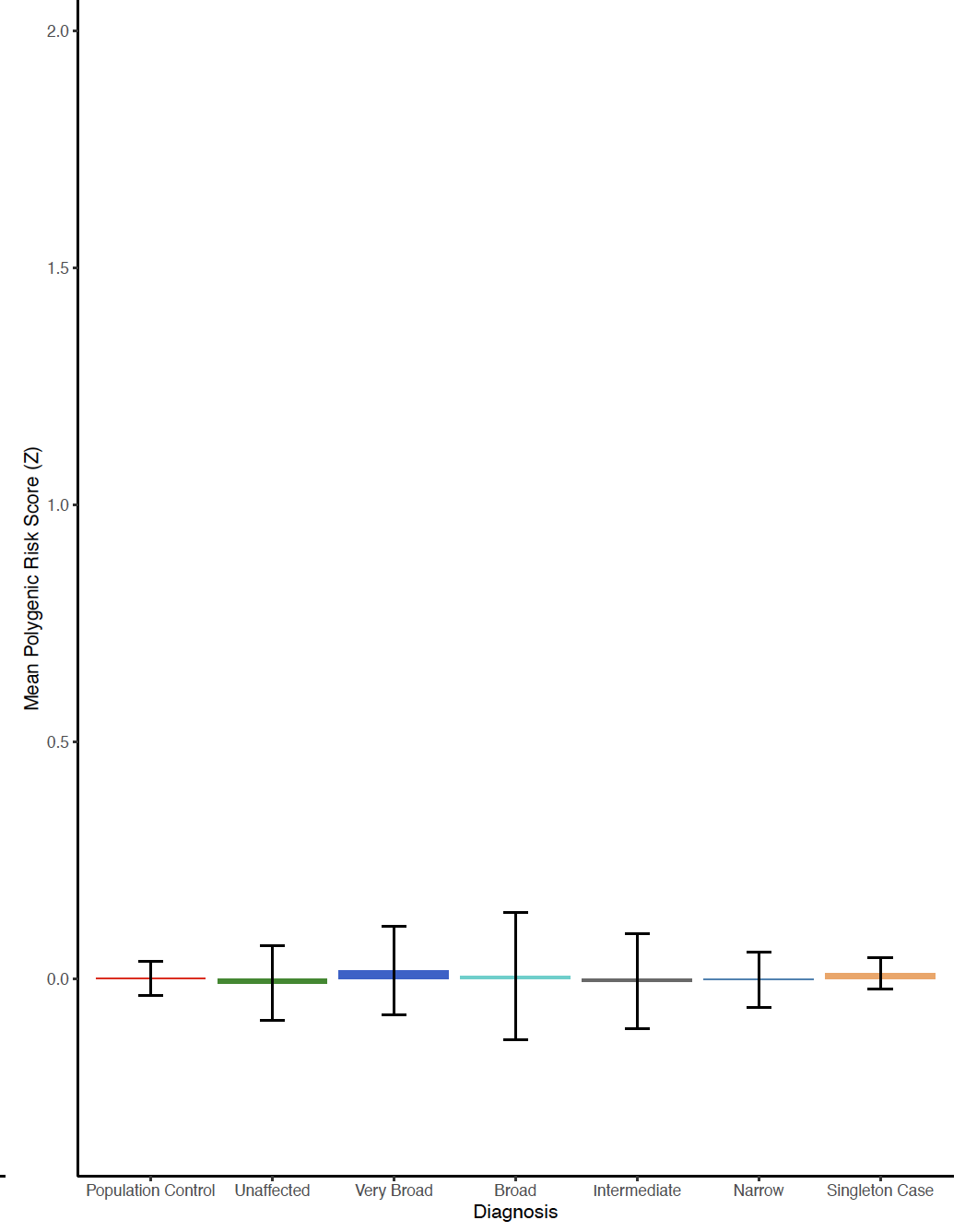


**Supplementary Figure 9:** Mean LDL PRS used as a negative control for each of the diagnostic categories in the ISHDSF sample, sporadic SCZ cases and ancestry-matched population controls. Error bars represent the standard error of the observed mean. X-axis shows each of the diagnostic categories. Y-axis shows the mean normalized Z-score for LDL.

| **Array** | **Total N** | **Controls** | **Singleton Cases** | **ISHDSF** | **N SNPs QC'd pre-imputation** | **N Imputed SNPs QC'd** |
| --- | --- | --- | --- | --- | --- | --- |
| **Illumina 610-Quad** | 830 | NA | NA | 830 | 557,373 | 9,298,012 |
| **Affymetrix V.60** | 1,731 | 1,731 | 1,509 | NA | 686,646 | 11,080,279 |
| **Infinium psychArray v.1.13** | 1,444 | 553 | 716 | 175 | 384,389 | 11,081,999 |

**Supplementary Table 2**: Description of the arrays used in this study. Number of individuals and pre/post imputation SNPs are provided.

| **Comparison Groups** | **OR** | **CI (95%)** | **P-value** | **Holm_adjusted_P** |
| --- | --- | --- | --- | --- |
| Sporadic Cases vs Control | 5.75 | 5.03-6.55 | 1.64E-48 | 3.78E-45 |
| Narrow vs Control | 5.95 | 5.07-6.93 | 1.18E-39 | 2.61E-33 |
| Intermediate vs Control | 4.17 | 3.33-5.11 | 1.54E-13 | 2.93E-11 |
| Broad vs Control | 3.96 | 3.05-4.90 | 3.72E-08 | 5.94E-7 |
| Very Broad vs Control | 3.38 | 2.46-4.38 | 6.12E-06 | 9.17E-5 |
| Unaffected vs Control | 2.55 | 1.84-3.16 | 2.75E-4 | 4.13E-3 |

**Supplementary Table 3:** Comparison results between diagnostic groups in the ISHDSF sample and sporadic cases versus population controls.

| **Comparison Groups** | **P-value** | **Holm_adjusted_P** |
| --- | --- | --- |
| Narrow vs Sporadic Cases | 0.29 | 0.90 |
| Narrow vs FH+ Sporadic Cases | 0.18 | 0.82 |
| Narrow vs FH- Sporadic Cases | 0.23 | 0.88 |
| FH+ vs FH- Sporadic Cases | 0.32 | 0.92 |
| Sporadic Cases vs Intermediate | 0.0006 | 0.0091 |
| Sporadic Cases vs Broad | 1.4E-5 | 5.9E-4 |
| Sporadic Cases vs Very Broad | 2.71E-5 | 6.1E-4 |
| Sporadic Cases vs Unaffected | 4.81E-15 | 7.61E-15 |
| Narrow vs Intermediate | 0.0004 | 0.008 |
| Narrow vs Broad | 3.5E-5 | 4.1E-4 |
| Narrow vs Very Broad | 1.87E-05 | 3.17E-4 |
| Narrow vs Unaffected | 5.73E-16 | 1.20E-14 |
| Intermediate vs Broad | 0.41 | 1 |
| Intermediate vs Very Broad | 1.1E-4 | 8.3E-3 |
| Intermediate vs Unaffected | 3.5E-5 | 4.6E-4 |
| Broad vs Unaffected | 0.0009 | 0.0083 |
| Broad vs Very Broad | 0.45 | 1 |
| Very Broad vs Unaffected | 0.46 | 1 |

Supplementary Table 4: Full comparison results for within ISHDSF analyses.

1. Das S, Forer L, Schönherr S, Sidore C, Locke AE, Kwong A, et al. Next-generation genotype imputation service and methods. Nat Genet. 2016;48(10):1284–7.

2. Purcell S, Neale B, Todd-Brown K, Thomas L, Ferreira MAR, Bender D, et al. PLINK: A Tool Set for Whole-Genome Association and Population-Based Linkage Analyses. Am J Hum Genet. 2007;81(3):559–75.

3. Auton A, Abecasis GR, Altshuler DM, Durbin RM, Bentley DR, Chakravarti A, et al. A global reference for human genetic variation. Vol. 526, Nature. 2015. p. 68–74.
